# Proteins of the SubB family provide multiple mechanisms of serum resistance in *Yersinia pestis*

**DOI:** 10.1080/22221751.2025.2493926

**Published:** 2025-04-16

**Authors:** François Pierre, Alexandre Baillez, Amélie Dewitte, Agustin Rolandelli, Florent Sebbane

**Affiliations:** Univ. Lille, CNRS, Inserm, CHU Lille, Institut Pasteur de Lille, U1019 – UMR 9017 – CIIL – Center for Infection and Immunity of Lille, Lille, France

**Keywords:** Plague, *Yersinia pestis*, serum resistance, SubB, SubB-Like

## Abstract

The serum complement system is a cornerstone element of the innate immune response. Bacterial resistance to this system is a multifaceted process involving various proteins and molecular mechanisms. Here, we report several genes required for the growth of *Yersinia pestis* in serum. Among them, we found that *ypo0337* encodes an outer-membrane-associated lectin that recruits factor H, C4BP and hemopexin, conferring resistance to the serum complement system. YPO0337 displays high sequence similarity with the SubB subunit of the AB_5_ toxin from *Escherichia coli*, as well as other SubB-like proteins, and *subB* from *E. coli* restores the ability of *Y. pestis* Δ*ypo0337* mutant to resist to serum complement. Altogether, the data suggest that at least two members of the SubB protein family function as virulence factors, conferring resistance to serum complement through a unique mode of action.

## Introduction

Cornerstone of the innate immune response, the serum complement system serves as one of the most potent mechanisms for the recognition and elimination of bacterial pathogens [1]. This system is activated through three main pathways – the classical, lectin, and alternative pathways that ultimately converge to facilitate opsonization, the release of pro-inflammatory signals that recruit immune cells to the site of infection, and the formation of the membrane attack complex (MAC), which causes bacterial cell lysis through the formation of membrane pores [2–6].

The classical and lectin pathway sets off a proteolytic cascade resulting in the formation of the C3 convertase [C4b2a], an enzyme complex that cleaves C3 into C3a and C3b [7–9]. C3b from spontaneous low-level hydrolysis of C3 in plasma [alternative pathway] can also bind directly to the bacterial surface [7,10]. Once fixed to the cells, C3b interacts with factor B to form the C3bBb complex, which acts as C3 convertase, cleaving additional C3 molecules to generate more C3b [7,11]. The accumulation of C3b on the pathogen surface promotes the formation of the C5 convertase complex (C4b2a(C3b)_n_ or C3bBb(C3b)_n_, depending on the pathway), which binds and cleaves the C5 compound into C5a and C5b. The later then associates with C6, C7, C8, and multiple C9 molecules to assemble the MAC (C5b-9 complex) [12–14].

While the serum complement system is essential for immune defence, a variety of inhibitors collectively ensure that the system operates effectively against pathogens while safeguarding the host’s own tissues from unintended damage [15]. Key regulators include factor H, which binds to C3b to promote its degradation and inhibit the alternative pathway [16]. This factor works in conjunction with factor I to inactivate C3b [17,18]. Interestingly, recent studies suggest that hemopexin, when associated with factor I, promotes the cofactor activity of factor I, facilitating the cleavage of C3b into iC3b, thereby aiding in complement regulation [19]. Key regulators of the serum complement system also include the C4BP, which inhibits the classical and lectin pathways of complement activation by binding and inhibiting the activity of the C4b fragment [20]. C4BP also interacts with C3b and mediates the decay of an alternative complement pathway [21].

The evasion to the serum complement system is a critical factor for bacterial survival and pathogenicity [22]. It is therefore not surprising that bacterial evolution led to the selection of an array of mechanisms that disrupt every stage of the complement cascade. Some bacteria (e.g. *Streptococcus*) produce a capsule that acts as a physical barrier, preventing complement deposition [23,24]. Other species (e.g. *Yersinia* and *Neisseria*) neutralize the serum complement system by recruiting host regulators, including factor H and C4BP, through protein–protein interactions or by expressing sialic acid on their surfaces [25-28]. Lastly, it has been reported that proteases are secreted by certain pathogens to cleave and inactivate complement proteins such as C3 and C5 [29,30].

We previously reported 10 *Y. pestis* mutants whose growth in serum differs from that of the wild-type strain [31]. However, we did not know whether the mutants’ growth defect was due to the bactericidal effect of the complement or a physiological consequence of the mutation. After assessing the mutants’ ability to grow in heat inactivated serum, we focused on the gene *ypo0337* which, during our investigation, was reported to encode a protein similar to the SubB subunit of the AB_5_ toxin and capable to bind sialic acid, promoting either host cell proliferation or death, depending on its amounts in the culture media [32,33]. Our data below indicate that YPO0337, along with at least one other member of the SubB protein family (SubB from *Escherichia coli*), functions as a new virulence factor that provides multiple mechanisms of serum resistance.

## Material and methods

### Bacteria and plasmids

Table S1 provides the bacteria and plasmids used in this study. Mutants were generated using the lambda Red recombinase and, when necessary, the I-*Sce*I selection system [34]. A *Y. pestis* strain in which the *ypo0337* coding sequence was replaced by the *subB* sequence from *E. coli* was also generated using the same methods. Table S2 provides relevant primers. Genotypes of the mutants were confirmed by PCR using primers flanking the target sequence outside the allelic exchange region, and sequencing was performed when necessary. Mutants were complemented with the recombinant pCRII plasmid.

### Bacterial survival in human serum

Normal serum was purchased from the Établissement Français du Sang (EFS), France’s public blood service. Serum was heat-inactivated at 56°C for 30 min [35], the alternative pathway was inactivated by incubation at 50°C for 20 min [36], and the classical and lectin pathways were inhibited using veronal buffer with 10 mM EGTA, 5 mM MgCl₂, and 0.1% gelatine [25]. Survival in serum was performed as previously described [31,37]. Briefly, *Y. pestis* cells cultured in Lysogeny Broth (LB) at 21°C were suspended in PBS at 4 × 10^7^ bacteria/mL. Then, 10 µL of the suspension was added to 90 µL of serum in 96-well plates and incubated at 37°C. Bacterial growth was measured by counting the CFUs or the brightness of the wells using Centro XS³ LB 960 luminometer (Berthold). Survival was assessed at 30, 60, and 180 minutes, and significant differences were observed after 60 minutes. However, the most pronounced difference between the wild-type and mutant strains occurred at 180 minutes, which is why we focused on this time point for our experiments. A statistically significant difference was also observed at 60 min.

### Protein purification and anti-YPO0337 antibodies

Sequences encoding YPO0337 or YPO0337 in which the serine at position 12 has been replaced by an Alanine (YPO0337-S12A) were cloned into the pET24d (+) vector at the *Nco*I and *Xho*I restriction sites. The c-term 6xHis tagged proteins were purified from *Escherichia coli* C41 cells using a cobalt resin according to the manufacturer’s instructions (Thermo Fisher). The following buffers were used during the workflow: binding/lysis denaturing buffer (PBS, 6 M guanidine-HCl, 10 mM imidazole, 0.05% (v/v) Triton X-100, EDTA-free protease inhibitors, 10% (v/v) glycerol, and 5 mM Mg-ATP); Wash Buffer 1 (25 mM Tris, 300 mM NaCl, 40 mM imidazole, 0.05% (v/v) Triton X-100); Wash Buffer 2 (25 mM Tris, 300 mM NaCl, 40 mM imidazole); and Elution Buffer (25 mM Tris, 300 mM NaCl, 500 mM imidazole, 0.05% (v/v) Triton X-100). The protein was further purified using a Hiload superdex 200pg high-resolution size-exclusion column (cytiva) using an Akta purifier chromatography system. Lastly, a HiTrap desalting column with Sephadex G-25 resin (cytiva) was used to exchange the buffer for PBS, and the soluble protein contained in PBS was used to generate antibodies by the Eurogentec company.

### Bioinformatics search and analysis

The UniProt database [38], Multiple Sequence Alignment (CLUSTALW) [39], membrane protein topology prediction (TMHMM) [40], and signal peptide prediction (SignalP-5.0) [41] were used with their default parameters to analyse the sequence of YPO0337.

### Detection of deposited serum components

The detection of serum components on the bacterial membrane was measured by Western-blot (C3b, C5b, C6, MAC, C4 BP, factor H and HPX) or ELISA (MAC) as previously described and using the antibodies provided in Table S3 [25,26,42]. Briefly, except for factor H, for which we used ∼1 × 10^9^ bacteria and heat-inactivated serum (50% final), ∼7.5 × 10^6^ bacteria were added to normal or heat-inactivated serum (90% final). Heat-inactivated serum was specifically used to eliminate complement activation and facilitate the detection of complement inhibitors. At the desired time points, samples were chilled on ice and then centrifuged at 4°C. For Western-blot analysis, the pellets were washed three times with chilled PBS, suspended in Laemmli buffer (Bio-Rad), loaded in a SDS-PAGE gel, and transferred to a nitrocellulose membrane using a Trans-Blot Turbo^TM^ blotting system with a pre-programmed protocol. After transfer, proteins were detected by an initial incubation with a primary antibody targeting the protein of interest (Table S3), followed by HRP-conjugated secondary antibodies. Detection was achieved using either SuperSignal West Pico (ThermoFisher) or Clarity Max ECL substrate (Bio-Rad), and images were captured with a Fujifilm LAS-3000 Luminescent Image Analyzer. Signal intensities were normalized using the RNA polymerase alpha subunit as a housekeeping protein [25,43]. For ELISA, bacterial cell pellets were washed twice with a buffer containing 137 mM NaCl, 2.7 mM KCl, 8.1 mM Na_2_HPO_4_, 1.5 mM KH_2_PO_4_, 1% glucose, 1.0 mM MgCl_2_, and 0.6 mM CaCl_2_. After washing, the pellets were dried by centrifugation, suspended in binding buffer (0.05 M NaHCO3, pH 9.6), and then equally divided into a 96-well plate (F16 MAXISORP Nunc-Immuno Module, Thermo Fisher) for either MAC detection or for standardization by Pla. After incubating the well contents with PBS containing BSA, proteins were detected by incubation with a primary antibody targeting the protein of interest (Table S3), followed by HRP-conjugated secondary antibodies. Detection was completed by adding TMB substrate (BioLegend) and stopping the reaction with 2N sulphuric acid, prior to measuring absorbance at 405 nm using an ELx800 microplate reader (BioTek).

### Localization of YPO0337 in the bacterial cell

*Y. pestis* WT, Δ*ypo0337* and the complemented mutant strain were subjected to cell fractionation using a detergent based and ultracentrifugation procedure to separate the cytoplasm, periplasm, outer, and inner membranes following cell lysis, as previously described [44]. Spheroplasts were generated by resuspending bacterial cell pellets in P buffer (0.2 M Tris-HCl pH 8, 1 M sucrose, 1 mM EDTA, 1 mg/mL lysozyme). After the addition of dH₂O and incubation at 4°C, the mixture was centrifuged at 200,000 × g for 45 min at 4°C to collect the supernatant containing the periplasmic fraction. The pellet was resuspended in ice-cold C buffer (10 mM Tris-HCl pH 7.5, 5 mM EDTA, 0.2 mM DTT, and 50 μL DNase at 1 mg/mL), and cells were lysed using a French press. The lysate was centrifuged at 4000 × g for 10 min at 4°C to remove unbroken cells and debris. The supernatant was then ultracentrifuged at 280,000–300,000 × g for 2–4 h at 4°C to collect the membrane fraction. The pellet was resuspended in IM buffer (50 mM Tris-HCl pH 8, 2% w/v Triton X-100, 10 mM MgCl₂) and ultracentrifuged at 85,000 × g for 30 min at 4°C to separate the membranes. The supernatant contained inner membrane proteins, while the pellet, containing outer membrane proteins, was washed and resuspended in dH₂O. After fractionation, the total protein concentration of each fraction was measured using the BCA method, and 10 µg of total protein per sample was used for western-blot analysis with the anti-YPO0337 antibody. After protein transfer and prior to incubation with the antibody, the membrane was stained with Ponceau to confirm loading and transfer accuracy.

### Identification of serum proteins interacting directly with YPO0337

TALON his-tag purification resin was mixed with purified His-tagged YPO0337. The resin was then divided into two equal portions: one portion was mixed with 50% normal serum (prepared by diluting 100% serum in 2X native binding buffer [50 mM Tris, 600 mM NaCl, 0.10% (v/v) triton X100, 80 mM Imidazol, pH 7.4]), and the other was mixed with 1X native binding buffer alone. After 2-hours incubation at 4°C, the resin was washed again as above, and the attached proteins were eluted with TNTI-500 elution buffer (25 mM Tris, 300 mM NaCl, 0.05% Triton X-100, 500 mM imidazole, pH 7.4). The proteins eluted with YPO0337 were separated by SDS-PAGE and visualized by comparison with the control (serum only) after Coomassie blue staining. The excised protein bands were processed under acidic conditions, washed with 100–200 µL of 20 mM ammonium bicarbonate buffer, and reduced with 1 mg/mL dithiothreitol (DTT) for 15 min. Alkylation was performed using 10 mg/mL iodoacetamide for 15–30 min. Gel bands were destained with a 1:1 mixture of 20 mM ammonium bicarbonate buffer and acetonitrile (3 × 10 min), then air-dried. Digestion was carried out overnight at 37°C using trypsin prepared in 1% acetic acid and diluted 50- to 100-fold in ammonium bicarbonate buffer. Extracted peptides were analysed by MALDI-TOF mass spectrometry in reflectron mode using a Bruker Autoflex Speed with an α-cyano-4-hydroxycinnamic acid (α-cyano) matrix. Data were processed with Bruker Daltonics Biotool (v3.2), and protein identification was performed via Swiss-Prot database searches. Their presence was further confirmed by western-blot using the antibodies listed in Table S3. The presence of protein that was not identified by SDS-PAGE but that we suspected to be eluted with YPO0337 (i.e. Factor H and C4BP) was also evaluated by western-blot.

## Results

### A refined list of *Y. pestis* genes needed for growth in serum due to a physiological/metabolic effect or effective resistance to complement

**Figure 1.**
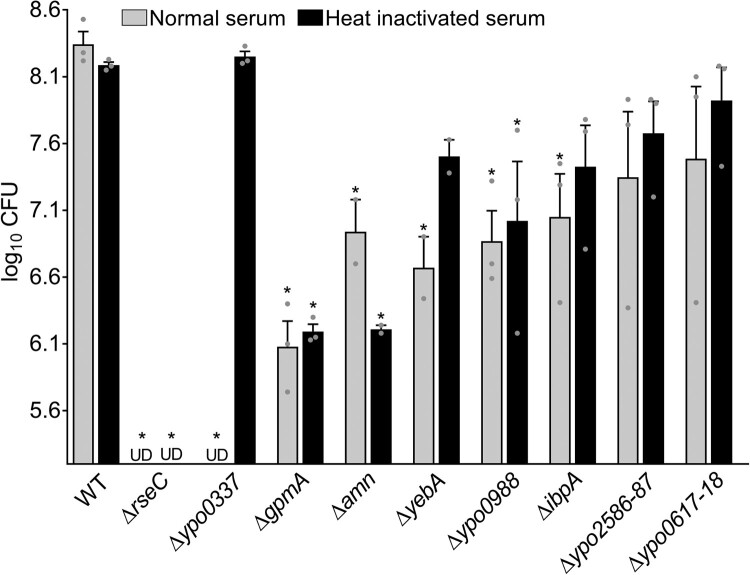
Role of *Y. pestis* genes in serum. The ability of previously selected mutants [31] to grow in normal and heat inactivated serum was evaluated after 21 h. The mean and standard error of the mean (SEM) of at least 2 independent experiments are shown, with each data point representing an individual replicate. *, *P* < 0.05 in a one-way analysis of variance. UD, undetected.

We previously listed *Y. pestis* mutants displaying growth attenuation in serum [31]. Here, we report that only the mutant lacking the *ypo0337* gene showed full restoration of growth when exposed to heat-inactivated serum (Figure 1). In other words, the data show that YPO0337 functions as a complement resistance factor whereas the characterized genes *rseC*, *gpmA*, *amn*, *yebA, ibpA* and the uncharacterized gene *ypo0988* provide a metabolic or physiological growth advantage to grow in serum. The role of the *ypo2586-2587* and *ypo0617-0618* loci in serum resistance may be host dependent.

### YPO0337 is an outer-membrane-associated protein that confers resistance to all three complement pathways via at least its serine at position 12

**Figure 2.**
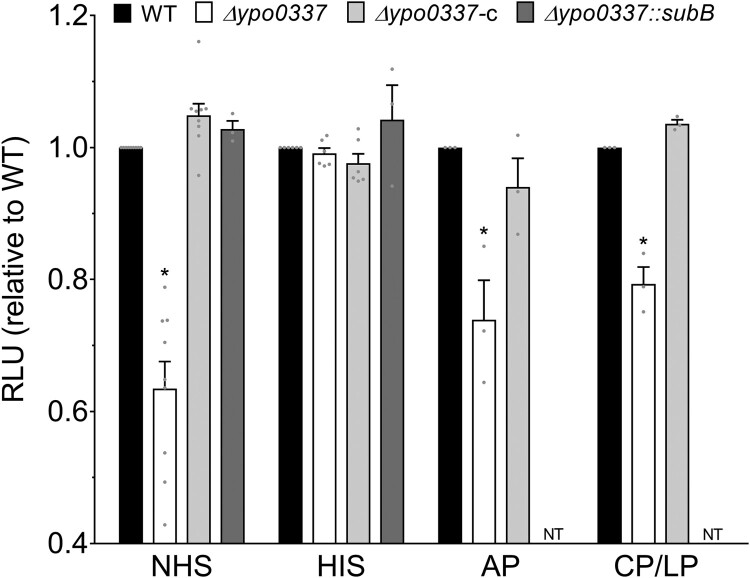
Role of YPO0337 in the resistance of *Y. pestis* to the different complement pathways. The survival of a bioluminescent *Y. pestis* wild-type strain (WT) (black bars), *Δypo0337* mutant strain (white bars) expressing a wild-type copy of *ypo0337* (grey bars) or in which *ypo0337* has been exchanged with *subB* from *E. coli* (dark grey bars) were determined after 180 min of contact with normal (NHS) or heat inactivated (HIS) serum, or with serum inactivated for alternative (AP) or the classical and lectin (CP/LP) pathways. The mean and SEM of at least 3 independent experiments (using sera from independent donors) and relative to the parental strain are shown, with each data point representing an individual replicate. *P < 0.05, in a one-way analysis of variance. NT, not tested.

**Figure 3.**
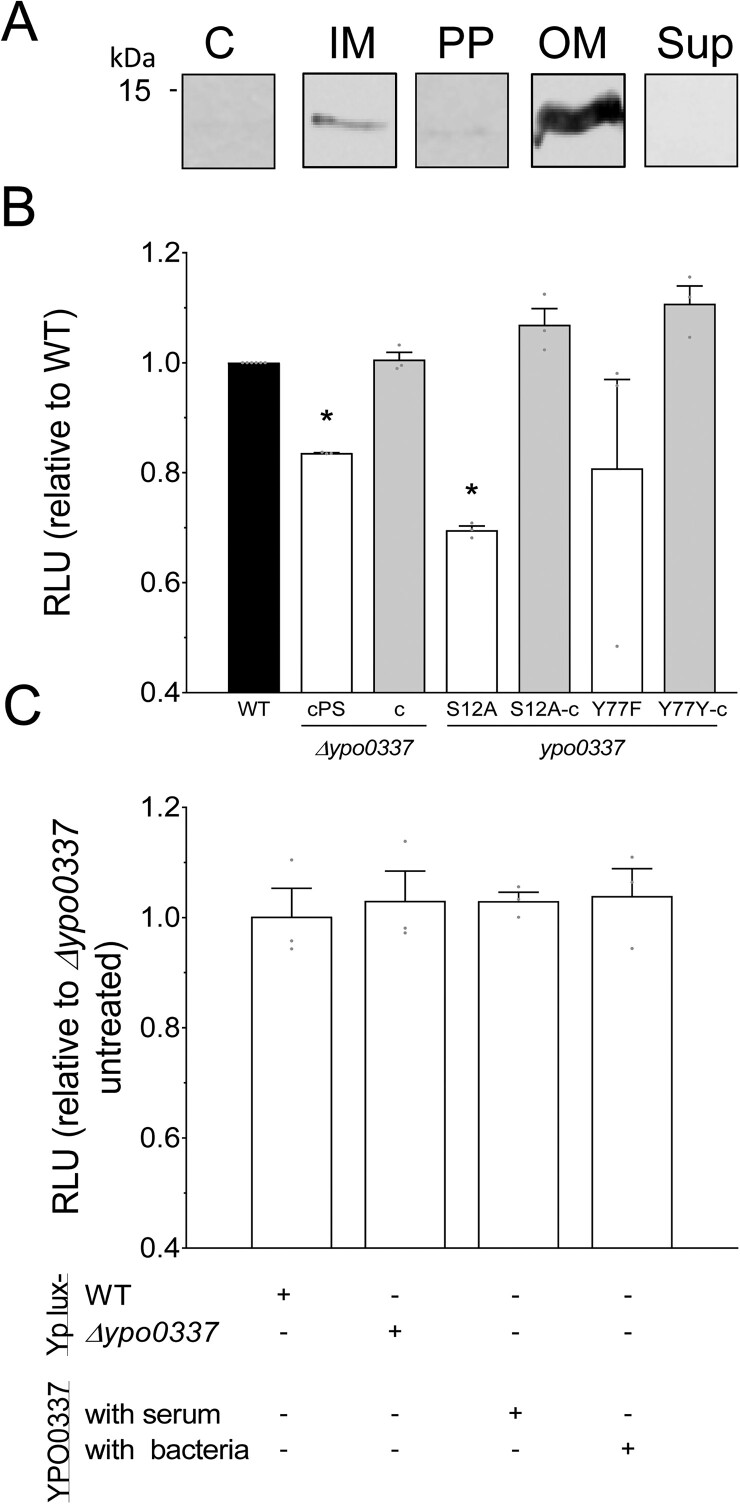
Cellular localization of YPO0337 and the role of its serine residue at position 12 in serum resistance. *A*, Immunoblot analysis of cell fractions (10 µg for each fraction) using an anti-YPO0337 antibody. Fractions include cytoplasm (C), inner membrane (IM), periplasmic (PP), outer membrane (OM), and culture supernatant (sup) from *Y. pestis* wild-type (WT) expressing YPO0337 from high copy number plasmid. Uncropped western-blot images used to generate the figure are provided in Figure S2. *B*, Survival of a bioluminescent *Y. pestis* strains after 180 min contact with normal serum. Strains includes wild-type (WT), a Δ*ypo0337* mutant producing YPO0337 without a signal peptide (cPS) or with it (c), as well as mutant strains with serine and tyrosine residues at positions 12 and 77 replaced by an alanine (S12A) and a phenylalanine (Y77F), respectively. The mean and SEM of 3 independent experiments (using sera from independent donors) and relative to the parental strain are shown, with each data point representing an individual replicate. *P < 0.05, in a one-way analysis of variance. *C*, The survival of a bioluminescent *Y. pestis* Δ*ypo0337* mutant strain was assessed after being mixed at a 1:5 ratio either with (+) or without (−) a non-bioluminescent *Y. pestis* (Yp lux-) wild-type (WT) strain, or a non-bioluminescent *Y. pestis* (Yp lux-) strain lacking YPO0337 (Δ*ypo0337*), followed by 180 min of contact with normal serum. Survival was also determined when 15 µg of purified YPO0337 was added (+) or not (−) either to the serum (“with serum”) or to the bioluminescent Δ*ypo337* mutant strain (“with bacteria”) prior to serum contact. Untreated strain refers to the bioluminescent Δ*ypo0337* mutant strain alone (not admixed with any other bacteria or protein), exposed to normal serum for 180 min. The mean and SEM of 3 independent experiments (using sera from independent donors) and relative to the untreated strain are shown, with each data point representing an individual replicate.

The Δ*ypo0337* mutant expressing a wild-type copy of *ypo0337* under its own promoter is fully resistant to normal serum, confirming that YPO0337 confers complement resistance (Figure 2). To determine whether this resistance is specific to individual complement pathways or extends to all three, we tested the mutant’s survival after inactivating the alternative (AP) or the classical and lectin (CP/LP) pathways. We found that the *ypo0337* mutant remained sensitive to serum when either AP or CP/LP pathways were inactivated, while the wild-type strain (WT) and complemented mutant were resistant, suggesting that YPO0337 provides broad protection against complement-mediated killing (Figure 2). This broad resistance points to a possible mechanism of action. Search for the location of YPO0337 using database, membrane topology, and signal peptide predictions suggested that YPO0337 is an exported protein that confers resistance through lectin activity. Indeed, YPO0337 has a signal peptide and shares ∼56% identity with the B subunit of the AB_5_ enterotoxins produced by Shiga-toxigenic *Escherichia coli* (STEC), a protein which binds sialic acid via its serine at positon 12 and tyrosine at position 78 (Figure S1). It was recently shown that YPO0337 exhibits broad specificity for sialic acid-terminated glycans and is a member of the SubB protein family, which binds sialic acids via a conserved serine [32,33]. Furthermore, it shares similar 3D structures with SubB from *E. coli* [32,33]. Consistent with the above hypothesis that YPO0337 is an exported outer-membrane-associated protein that confers resistance through a lectin activity, western-blot analysis revealed that YPO0337 (detected only when overproduced by the bacterium) was primarily present in the outer membrane (Figure 3(A)). Furthermore, a *Y. pestis* strain producing YPO0337 lacking of its signal peptide or in which the serine 12 has been replaced by alanine (S12A) grew in serum only when complemented with a wild-type copy of *ypo0337* or when the serum was inactivated (Figure 3(B)). In contrast, a mutant YPO0337-Y77F (i.e. lacking the conserved tyrosine involved in sialic acid binding) was as resistant as the WT strain to the serum complement, indicating that the binding of sialic acid via the serine at position 12 is solely required for serum resistance. Lastly, co-culturing the Δ*ypo0337* mutant with the WT strain or in the presence of purified YPO0337 in serum did not rescue the mutant’s defect, suggesting that YPO0337 is not secreted in the media to inhibit the action of the complement (Figure 3(C) and S5). Altogether, the data show that YPO0337 specifically acts at the outer membrane, presumably as a lectin, and confers serum resistance via its Ser12 by affecting all complement pathways.

### YPO0337 inhibits the fixation of several compounds of the complement through recruiting complement inhibitors

**Figure 4.**
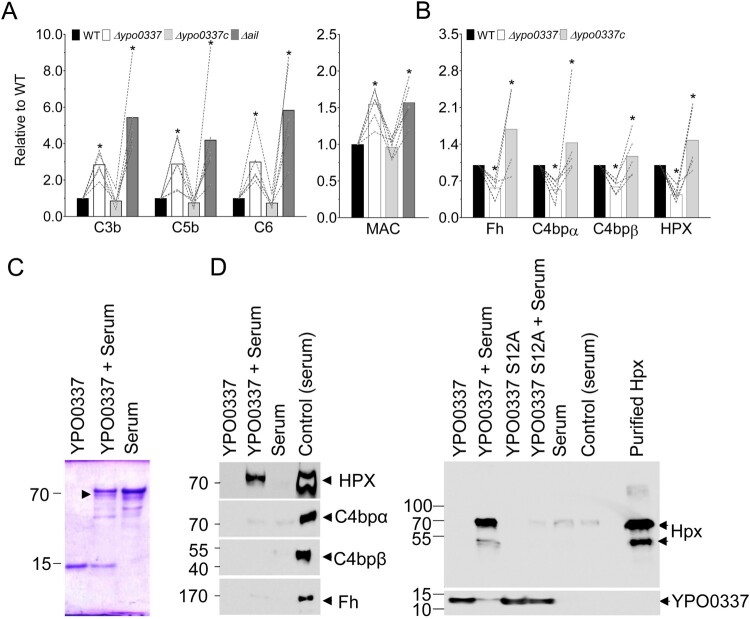
Impact of YPO0337 on the deposition of complement components on the bacterial surface. *A-B*, Immunoblot analysis showing activated complement components (C3b, C5, C6) or complement inhibitors (Fh, C4bpα, C4bpβ, HPX) and ELISA analysis showing MAC complex bound to the wild-type strain (WT), Δ*ypo0337*, complemented Δ*ypo0337* (Δ*ypo0337c*) and Δ*ail* mutant (used as a control) after contact with normal (*A*) or heat inactivated serum (*B*). Heat-inactivated serum was specifically used in (B) to avoid complement activation and facilitate the detection of complement inhibitors without interference from activated complement components, which are shown in (A) using normal serum. Data represent the ratio of C3b, C5 and C6 to RNA polymerase and MAC to Pla relative to WT. The data using at least 4 different sera are shown. Each line connects the data obtained with a given serum. *P < 0.05, in a one-way analysis of variance. *C*, Coomassie blue-stained SDS-PAGE showing a unique serum protein (arrow) co-purified with His-tagged YPO0337 in a pull-down experiment (YPO0337 + Serum lane). Mass spectrometry identified the protein as Hemopexin (Hpx). Controls includes pulled down using only the His-tagged YPO0337 or the serum. *D*, Western-blot analysis of proteins from the pull-down experiment (i.e. from C) using anti-Hpx, anti-C4BP (α and β), anti-Fh and anti-YPO0337 antibodies. In comparison to C, the serum alone (Control (serum)) or the purified Hpx was used as control to detect the proteins of interest. Representative uncropped images used to generate panels A, B, and D are provided in Figures S3 and S4.

To determine how YPO0337 inhibits the complement cascade and identify its point of action, we assessed the deposition of C3b, C5, C6, and MAC on the bacterial surfaces using Western-blot and ELISA, and using the Δ*ail* mutant as a control since Ail is known to interfere with the cascade of the complement [25] (Figure 4). When comparing the data obtained with the WT strain and the Δ*ypo0337* strain complemented or not by a wild-type copy of *ypo0337*, our results showed that YPO0337 deficiency led to increased levels of these complement components, indicating that YPO0337 plays an early role in the cascade by recruiting complement inhibitors (Figure 4(A)). Supporting this, several lines of evidence suggest that YPO0337 inhibits the alternative pathway by promoting factor H binding, an early complement inhibitor. First, the YPO0337-S12A mutant is unable to resist complement (Figure 3(B)). Second, the Serine residue at position 12 is known to be critical for binding sialic acid by the proteins of the SubB protein family [32,33]. Lastly, sialic acid facilitates factor H recruitment to the cell surface and factor H contains sialic acid in its glycans [28,45]. Consistent with this, the YPO0337 mutant exhibited reduced factor H binding compared to the WT and complemented strains (Figure 4(B)). While evidence exists for how YPO0337 inhibits the alternative pathway, the mechanisms for classical and lectin pathway inhibition remain unclear. To address this, we examined the recruitment of C4BP and found that the YPO0337 mutant was deficient for C4BP recruitment (Figure 4(B)). In conclusion, our findings suggest that YPO0337 recruits both factor H and C4BP, thereby inhibiting all three complement pathways.

### YPO0337 recruits hemopexin in addition to factor H and C4BP

To confirm the recruitment of factor H and C4BP by YPO0337 and investigate how YPO0337 interferes with the complement cascade, we further performed a pull-down assay using purified YPO0337 and normal serum, followed by mass spectrometry to identify the interacting proteins. Unexpectedly, C4BP and Factor H were not identified by this approach. A pull-down assay followed by a western-blot targeting these proteins clarified the result. The blot revealed that C4BP could not be distinguished from the controls due to its non-specific binding to the chromatography column used for the purification of YPO0337 and its partners (Figure 4(D)). In the case of Factor H, its absence was likely due to its extremely low yield during purification, as the blot barely detected it (Figure 4(D)). Last but not least, the pull-down assay followed by mass spectrometry identified only one relevant serum protein, hemopexin (Figure 4(C) and Table S4), which was confirmed by the pull-down assay followed by western-blot (Figure 4(D)). Hemopexin is a plasma glycoprotein bearing sialic acid residues, found in serum as complex with Factor I, and participating in the inhibition of the complement cascade [19,46]. We further found that the Δ*ypo0337* mutant exhibited reduced hemopexin binding compared to the WT and complemented strains (Figure 4(B)). Interestingly, hemopexin was not detected in pull down assays when using the YPO0337-S12A version (Figure 4(D)). Together, our findings suggest that YPO0337 recruits Factor H and hemopexin via their sialic acid residues, with Ser12 of YPO0337 playing a key role in this recruitment. Moreover, they also show that YPO0337 recruits C4BP through a mechanism that remains unclear. Lastly, our data show that these interactions enhance serum resistance by interfering with the three pathways of the complement system.

### SubB from *E. coli* rescues the absence of YPO0337 in *Y. pestis*

In *E. coli*, s*ubB*, which together with *subA* forms an operon, encodes the B subunit associated with the A subunit of the SubAB_5_ toxin [47]. In *Y. pestis*, YPO0337 is not part of an operon and there is no *subA*-like gene, indicating that YPO0337 encodes an orphan B subunit (Figure S6). Therefore, although SubB from *E. coli* and YPO0337 from *Y. pestis* are structurally related, we wondered whether *E. coli* SubB might have the same function as YPO0337 with respect to serum resistance. To address this question, we measured the survival in serum of a YPO0337 mutant expressing the *E. coli* SubB protein, as well as when the bioluminescent *Y. pestis* Δ*ypo0337* mutant was co-cultured with a *Y. pestis* strain in which *ypo0337* was replaced by *subB*. The results show that the strain complemented by *subB* survived as well as the WT strain in normal serum (Figure 2), while the presence of the strain expressing *subB* did not rescue the bioluminescent mutant’s defect (Figure S5). Together, these findings demonstrate that YPO0337 is functionally interchangeable with SubB from *E. coli*. Thus, our data highlight that the SubB family of proteins may constitute a new family of complement resistance factors.

## Discussion

Here, we report that YPO0337 is an outer-membrane-associated protein conferring resistance to all three complement pathways by recruiting factor H, C4BP, and hemopexin to the bacterial surface (Figures 1–4). This recruitment likely involves sialic acid recognition, given that (1) factor H, C4BP, and hemopexin all contain sialic acid in their glycans [46,48,49], and (2) YPO0337 belongs to the SubB protein family, which is known for binding sialic acid [32,33]. In this family, a conserved serine residue enables recognition of various sialic acids. Accordingly, we showed that the YPO0337-S12A variant – lacking a conserved serine residue – fails to confer complement resistance (Figure 3). Altogether, our findings support a model where YPO0337 acts as an outer-membrane-associated lectin, recruiting factor H, C4BP, and hemopexin via sialic acid binding to inhibit all complement pathways. As such, YPO0337, in addition to Ail [25], is the second factor needed for serum resistance in *Y. pestis* and appears to be the prototype of a new bacterial serum resistance factor.

Assuming YPO0337 attracts complement inhibitors through sialoglycan recognition, it might also recruit additional inhibitors not examined in this study, such as C1 inhibitor, clusterin, and vitronectin, which block various complement pathways and contain sialic acid in their glycans [48,50,51]. If YPO0337 can recruit multiple inhibitors, the most abundant may be preferentially recruited, explaining why hemopexin, but not the less abundant factor H, was prominently identified in YPO0337 pull-down experiments (Figure 4). YPO0337 may also recruit factor H for mimicking host or pathogenic mechanisms, as observed in *Neisseria*, which recruits factor H via sialic acid binding for immune evasion [28].

While investigating the role of YPO0337 in the pathogenesis of plague, another group identified YPO0337 (aka YpeB) as a lectin with the ability to bind sialic acids [33]. They further showed that YPO0337’s lectin-like activity can stimulate host cell proliferation, and, if provided in sufficiently high quantities in the culture medium, can even induce cell death. However, it is noteworthy that the above functions were observed at concentrations of 0.5–0.6 µg/mL of purified YPO0337 (i.e. approximately 2.10^13^ molecules/mL). If YPO0337 is as abundant as the outer membrane protein Lpp, with approximately 10^6^ molecules per cell [52,53], this concentration would equate to about 2.10^7^ bacteria/mL – levels observed only at advanced disease stages [54]. However, we detected YPO0337 only when overexpressed from a high-copy plasmid (Figure 3(A)), indicating that YPO0337 is not abundantly produced, unlike Lpp. Additionally, YPO0337 does not seem to be secreted into the environment or at least in significant amounts. Thus, it is unlikely that YPO0337 naturally promotes cell proliferation or cell death *in vivo*. Further studies using physiological concentrations of YPO0337 or a Δ*ypo0337* mutant *in vivo* are needed to clarify the role of YPO0337 in cell proliferation *in vivo*. Nevertheless, our overall data indicate that the primary physiological role of *Y. pestis* YPO0337 is likely to confer serum complement resistance.

YPO0337 shares ∼56% identity with the SubB subunit of the secreted cytotoxin subtilase SubAB produced by Shiga toxigenic *E. coli* strains. SubAB belongs to the AB_5_ toxins which are important virulence factors produced by several pathogens [32,33,47]. Surprisingly, YPO0337 appears to be orphan SubB-like subunit. Comparative genomic analyses and experimental observations have revealed that pathogenic bacterial species (*E. coli*, *Salmonella enterica* serovar Typhi and *S. typhimurium*) produce a SubB-like protein that binds to sialic acid via a conserved serine residue [32,33]. Here, we report that *E. coli* SubB attenuates the absence of YPO0337 and that the conserved serine in YPO0337 is required for serum resistance. Therefore, we suggest that the entire SubB protein family, or at least several members within this family, functions as a virulence factor shared by many pathogens to resist serum complement.

## Supplementary Material

Pierre_Supplementary_Tables and figures_R2.pdf
