# Supplementary material for: Proteins of the SubB family provide multiple mechanisms of serum resistance in Yersinia pestis
Source: Emerg Microbes Infect. 2025 Apr 16;14(1):2493926. doi: 10.1080/22221751.2025.2493926 (PMC12064104; doi:10.1080/22221751.2025.2493926)
Supplement: Pierre_Supplementary_Tables and figures_R2.pdf [file TEMI_A_2493926_SM7973.pdf]

Supplementary Table 1. Strains and Plasmids used in the study.

Strains and & plasmids	Relevant characteristics	References
Y. pestis		
CO92	pYV−positive strain, virulent strain	[31]
CO92 *ΔrseC*	Km^R	[31]
CO92 *Δypo0337*	Km^R	[31]
CO92 *ΔgpmA*	Tp^R	[31]
CO92 *Δamn*	Zeo^R	[31]
CO92 *ΔyebA*	Tp^R	[31]
CO92 *Δypo0988*	Km^R	[31]
CO92 *ΔibpA*	Tp^R	[31]
CO92 *Δypo2586-87*	Zeo^R	[31]
CO92 *Δypo0617-18*	Km^R	[31]
KIM6+	pYV−negative strain, attenuated strain	[31]
KIM6+ Lux+	Bioluminescent strain	[34]
KIM6+ Lux+ Δail	Km^R	Laboratory collection
KIM6+ *Δypo0337*	Km^R	This work
KIM6+ *Δypo0337::subB*	*ypo0337* exchanged by *subB* gene	This work
KIM6+ Lux+ pEP1346	harboring pEP1346, lux+, Ap^R	This work
KIM6+ *Lux+ Δypo0337*	lux+, Km^R	This work
KIM6+ Lux+ Δ*ypo0337*-c	harboring pFP1, lux+, Km^R Ap^R	This work
KIM6+ Lux+ Δ*ypo0337*::*subB*	*ypo0337* exchanged by *subB* gene, lux+	This work
KIM6+ Lux+ Δ*ypo0337*-cPS	harboring pFP2, lux+, Km^R Zeo^R	This work
KIM6+ Lux+ *ypo0337*::S12A	*ypo0337* exchanged by *ypo0337* gene with serine 12 substitution, lux+	This work
KIM6+ Lux+ *ypo0337*::S12A-c	harboring pFP1, lux+, Km^R Ap^R	This work
KIM6+ Lux+ *ypo0337*::Y77F	*ypo0337* exchanged by *ypo0337* gene with tyrosine 77 substitution, lux+	This work
KIM6+ Lux+ *ypo0337*::Y77F-c	harboring pFP1, lux+, Km^R Ap^R	This work
E. coli		
DH5α	*F- Φ80lacZΔM15 Δ(lacZYA-argF) U169 recA1 endA1 hsdR17 glnV44 thi-1 gyrA96 relA1 Δ(lacZYA-argF)U169 zdg-232::Tn10*	Invitrogen
C41	derived from Bl21(DE3) ; *F– ompT gal dcm hsdSB(rB- mB-)*	Laboratory collection
O157:H7	source of *subB*	Laboratory collection
Plasmid		
pCRII	cloning vector, Km^R, Ap^R	Invitrogen
pCR blunt	cloning vector, Km^R, Zeo^R	Invitrogen
pET24d +	T7 promoter, *pelB* , pBR322 origin, *aph* (3')-II (kanR2) , *lacI* , C-terminal His_6, expression vector	Adgene
pEP1436	vector containing the Red recombinase *(arabinose unductible)* and the endonuclease I-*Sce* I (doxycycline inductible), Ap^R	[34]
pEP1446	from pKD4; containing the kanamicyn resistant gene and I-*Sce* I sequence	[34]
pFP1	pCRII containing *ypo0337* gene under control of its own promotor	This work
pFP2	pCR-blunt containing *ypo0337* gene under control of its own promotor, but without signal peptide sequence	This work
pFP3	pET24d+ ; *ypo0337* under control of arabinose promotor and start codon present in the *NcoI* sequence	This work
pFP4	pET24d+ ; *ypo0337::S12A* under control of arabinose promotor and start codon present in the *Nco* I sequence	This work

Supplementary Table 2. Primers used in the study.

Primers	Sequences	
ypo0337 -Ext F	TACCTTTTGACATCCCGGCC	verification and complementation
ypo0337 -Ext R	CGCGCGCAAAACTATCTGCT	verification and complementation
ypo0337 -Up (*NcoI)*	CATG*CCATGG* CGCGTTATTTATTATCTCTGTC	expression Primer
ypo0337 -Down (*XhoI*)	CCG*CTCGAG* ATGCACGGTAAATTTATGTT	expression Primer
Mut-*ypo0337* -Up	**TAATAGAACAGTATTTTTTCATGTGTCATTGGAGGGTAAAGTATG**GTGTAGGCTGGAGCTGCTTC	generate the mutant
Mut-*ypo0337* -Down	**CATTGGCGGGTTCGTTGACATTAAAAGGAAACATTCACTTTAATG**CATATGAATATCCTCCTTAG	generate the mutant
ypo0337 ::S12A-Up1	**GCATTTGCTGAATGGACTGGTGATAACGTAGAAGGTATGCAT**G**CA**GTGTAGGCTGGAGCTGCTTC	generate the mutant
ypo0337 ::S12A-down1	**TTTCCCATCAACCTGGCCTGAATGAAATTTATTTATAATCATGCC**CATATGAATATCCTCCTTAG	generate the mutant
ypo0337 ::S12A-Up2	**GCTGAATGGACTGGTGATAACGTAGAAGGTATGCAT**G**CAGGCATGATTATAAATAAATTTCATTCAGGCCAGGTTGAT**	generate the mutant
ypo0337 ::S12A-down2	**ATCAACCTGGCCTGAATGAAATTTATTTATAATCATGCCTG**C**ATGCATACCTTCTACGTTATCACCAGTCCATTCAGC**	generate the mutant
ypo0337 ::Y77F-Up1	**GCTATGTATTATTATACAACCGGTAAGCGTATCAGAGTTTATT**T**C**GTGTAGGCTGGAGCTGCTTC	generate the mutant
ypo0337 ::Y77F-down1	**TGTAAGTGCCCTCACAAAACTATTATTAGTCCAGACATCCGGAGC**CATATGAATATCCTCCTTAG	generate the mutant
ypo0337 ::Y77F-Up2	**TATTATTATACAACCGGTAAGCGTATCAGAGTTTATT**T**CGCTCCGGATGTCTGGACTAATAATAGTTTTGTGAGGGCA**	generate the mutant
ypo0337 ::Y77F-down2	**TGCCCTCACAAAACTATTATTAGTCCAGACATCCGGAGCG**A**AATAAACTCTGATACGCTTACCGGTTGTATAATAATA**	generate the mutant
ypo0337 ::*SubB* -up	**TAATAGAACAGTATTTTTTCATGTGTCATTGGAGGGTAAAGTATG**ACGATTAAGCGTTTTTTTGT	generate the mutant
ypo0337 ::*SubB* -down	**CATTGGCGGGTTCGTTGACATTAAAAGGAAACATTCACTTTAATG**TTATGAGTTCTTTTTCCTGTCA	generate the mutant
ypo0337 -Ov-R	CTTCTACGTTATCACCAGTCCATTC CATACTTTACCCTCCAATGA	overlap PCR - Psmut
ypo0337 -Ov-F	GAATGGACTGGTGATAACGTAGAAGGTATGCATTCAGGCATGATT	overlap PCR - Psmut
Kan - F	CGGTGCCCTGAATGAACTGC	verification
Kan - R	AGGCTATTCGGCTATGACTG	verification

black = *Yersinia pestis* sequences; italic = restriction sequences; underline = *ypo0337* sequences; bold character = recombination sequences; Blue and green = kanamycin sequences; red = substitution base; orange = *subB* sequences

Supplementary Table 3. Antibodies used in the study.

name	clonality	specie	reaction	dilution	clone	supplier
C3b	monoclonal	mouse	human	1/2000	B-9	Santa cruz Biotechnology
C5b	monoclonal	mouse	human	1/2000	E-8	Santa cruz Biotechnology
C6	monoclonal	mouse	human	1/900	D-8	Santa cruz Biotechnology
HPX	monoclonal	mouse	human	1/5000	F-12	Santa cruz Biotechnology
FH	monoclonal	mouse	human	1/900	C18/3	Santa cruz Biotechnology
C4bpα	monoclonal	mouse	human	1/1000	D-5	Santa cruz Biotechnology
C4bpβ	monoclonal	mouse	human	1/900	E-1	Santa cruz Biotechnology
MAC	monoclonal	mouse	human	1/500	aE11	Santa cruz Biotechnology
Pla	monoclonal	mouse	*Y. pestis*	1/1500	Pla35	CEA
RNA pol	monoclonal	mouse	*E. coli*	1/5000 (10^6 bacteria)	4RA2	Biolegend
RNA pol	monoclonal	mouse	*E. coli*	1/50000 (10^9 bacteria)	4RA2	Biolegend
YPO0337	polyclonal	rabbit	*Y. pestis*	1/900		This study
Anti-mouse	polyclonal	goat	mouse	1/5000		Invitrogen
Anti-rabbit	polyclonal	goat	rabbit	1/5000		Invitrogen

Supplementary Table 4. Mass spectrometry identification of human serum proteins interacting with YPO0337.

Protein[1]	Score	Number of peptides detected	Intensity coverage	Sequence coverage	pI	MW (Kda)	mass of peptide (m/z)	Sequence of the detected peptide
Human Albumin[2]	98,3	12	39,60%	25%	5,9	71,3	875,51	LSQRFPK
							927,49	YLYEIAR
							960,56	FQNALLVR
							1074,54	LDELRDEGK
							1149,58	DAHKSEVAHR
							1467,83	RHPDYSVVLLLR
							1639,94	KVPQVSTPTLVEVSR
							1910,94	RPCFSALEVDETYVPK 3: Carbamidomethyl (C)
							1931,99	SLHTLFGDKLCTVATLR 11: Carbamidomethyl (C)
							2045,1	VFDEFKPLVEEPQNLIK
							1445,21	EFNAETFTFHADICTLSEKER 14: Carbamidomethyl (C)
							2599,27	QNCELFEQLGEYKFQNALLVR 3 : Carbamidomethyl (C)
Human hemopexin	77	9	26,80%	22,50%	6,6	52,4	813,44	LHIMAGR 4: Oxidation (M)
							828,42	SHKWDR
							1070,57	GEVPPRYPR
							1142,6	QGHNSVFLIK
							1220,6	NFPSPVDAAFR
							1268,66	FDPVRGEVPPR
							1495,67	YYCFQGNQFLR 3: Carbamidomethyl (C)
							2364,18	LLQDEFPGIPSPLDAAVECHR 19: Carbamidomethyl (C)
							2498,27	EVGTPHGIILDSVDAAFICPGSSR 19: Carbamidomethyl (C)

[1], The listed proteins were identified from the band excised for mass spectrometry analysis, as indicated by the arrow in Figure 4C. The band was selected by visual comparison with the control (serum only)

[2], Albumin was considered a contaminant due to its high abundance in serum and its well-documented tendency to bind nonspecifically to surfaces in proteomic experiments.

Figure S1

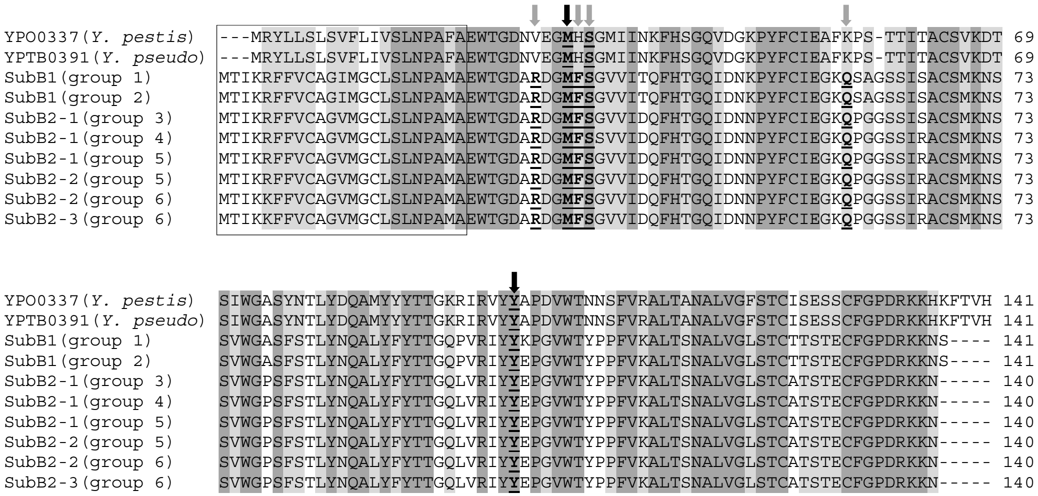

Figure S1. Sequence alignment of YPO0337 from *Yersinia pestis*, YPTB0391 from *Yersinia pseudotuberculosis*, and SubB family proteins from distinct *E. coli* strains. Identical and similar amino acids are highlighted in dark and light grey respectively. Bold letters indicate residues required for sialic acid binding. Grey arrows indicate residues required for NeuAc and Neu5GC binding, while black arrows indicate residues involved only in Neu5Gc binding. Boxed residues correspond to the signal peptide sequence.

Figure S2

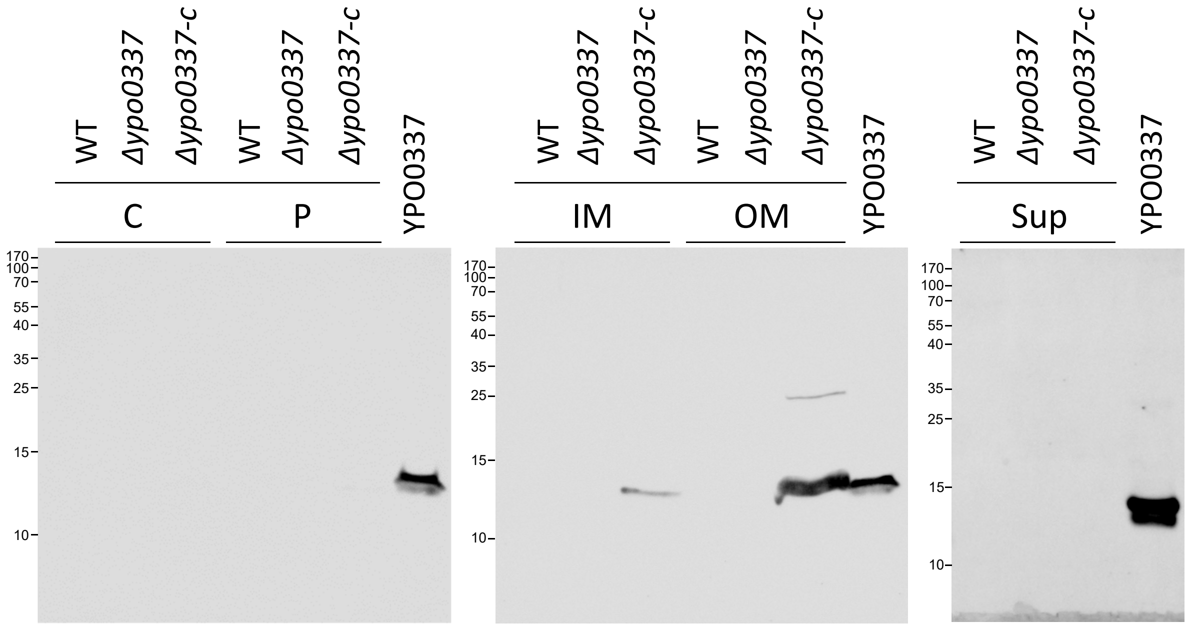

Figure S2. Uncropped western-blot images used to generate Figure 3A. Numbers next to the gels indicate protein molecular weight (kDa).

Figure S3

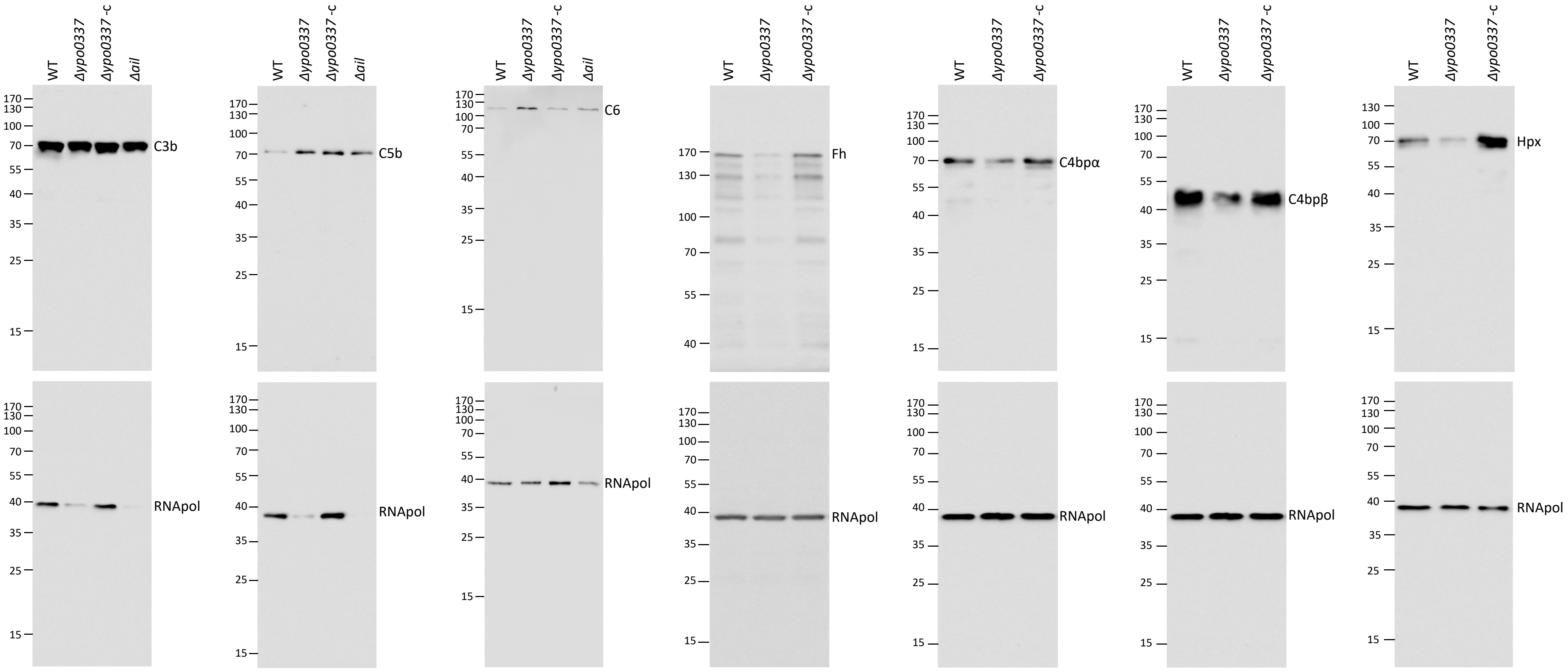

Figure S3. Uncropped representative western-blot images used to generate figures 4A and 4B. Numbers next to the gels indicate protein molecular weight (kDa).

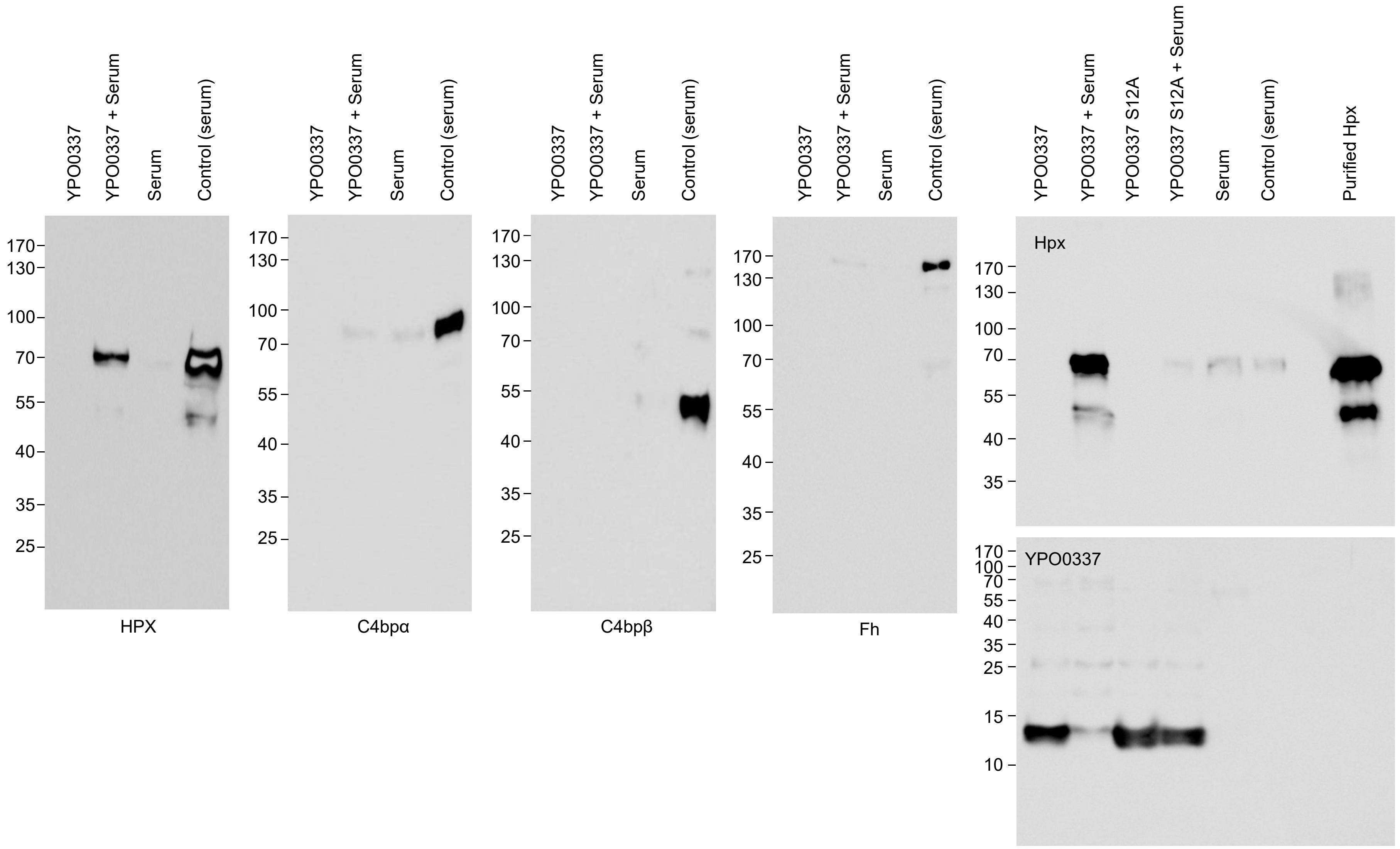

Figure S4. Uncropped western blot images used to generate Figure 4D. Numbers next to the gels indicate protein molecular weight (kDa).

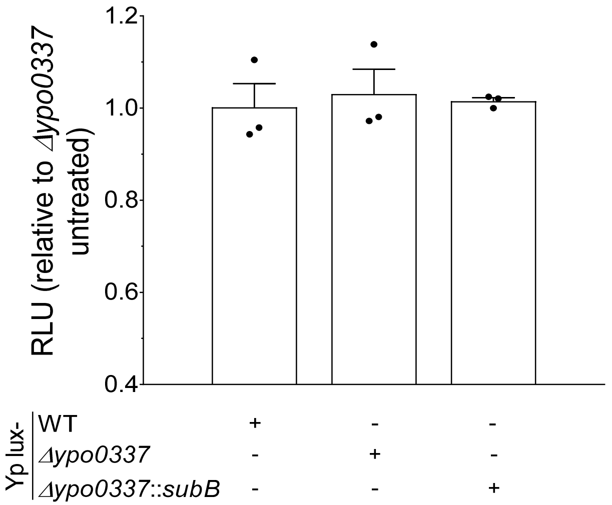

Figure S5. The survival of a bioluminescent *Y. pestis* Δ*ypo0337* mutant strain was assessed after mixing with a non-bioluminescent *Y. pestis* (Yp lux-) wild-type (WT) strain, a non-bioluminescent *Y. pestis* (Yp lux-) strain lacking YPO0337 (Δ*ypo0337*), or , a non-bioluminescent *Y. pestis* (Yp lux-) in which *ypo0337* has been exchanged with *subB* from *E. coli* (Δ*ypo0337*::*subB)* at a 1:5 ratio, followed by 180 minutes of contact with normal serum. The mean and SEM of 3 independent experiments (using sera from independent donors) and relative to the untreated strain are shown, with each data point representing an individual replicate used to generate the bars and SEM.

Figure S6

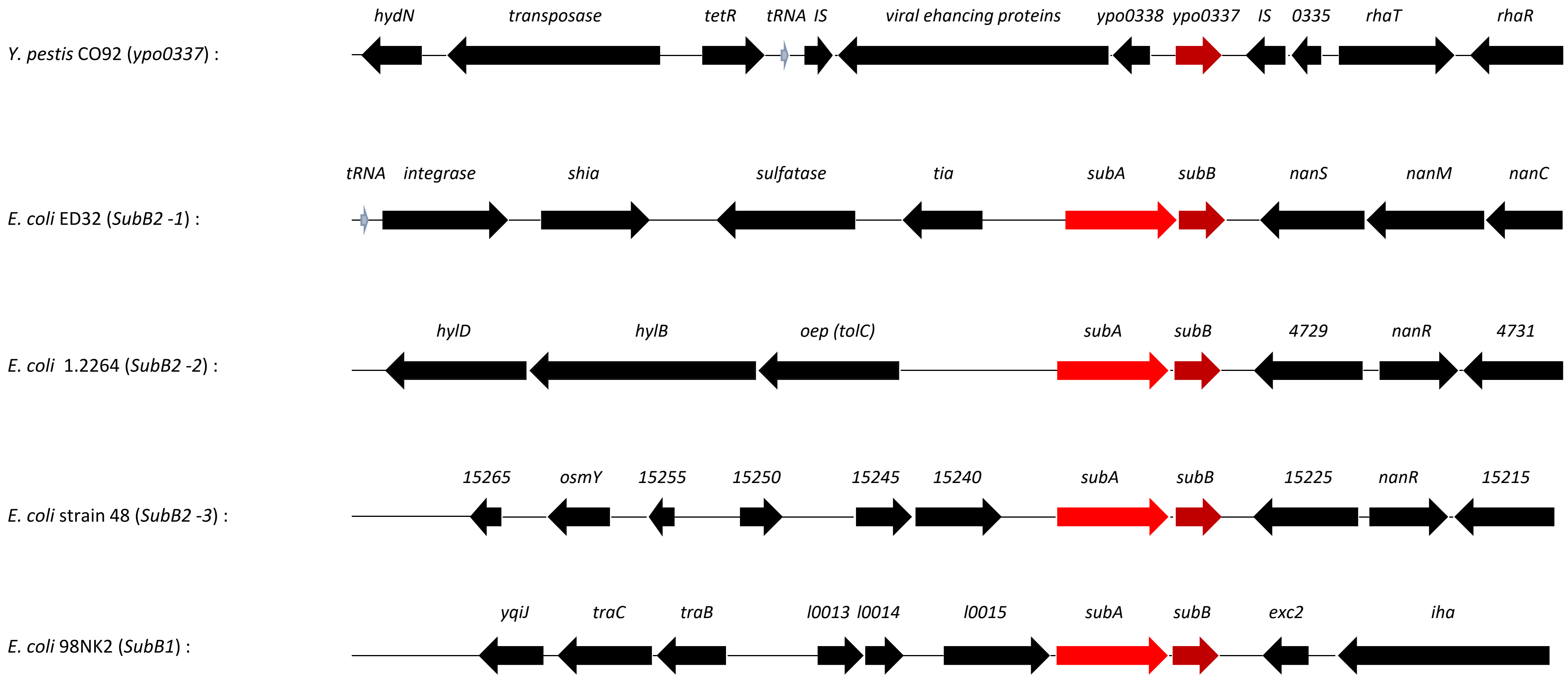

Figure S6. Genetic organization of the immediate environment of *ypo0337* and *subB.*
